# Do size, shape, and alignment parameters of the femoral condyle affect the trochlear groove tracking? A morphometric study based on 3D- computed tomography models in Chinese people

**DOI:** 10.1186/s12891-016-1374-3

**Published:** 2017-01-06

**Authors:** Zhe Du, Shichang Chen, Mengning Yan, Bing Yue, Yiming Zeng, You Wang

**Affiliations:** 1 Department of Orthopaedic Surgery, Renji Hospital, School of Medicine, Shanghai Jiaotong University, 145 Shandong Middle Road, Shanghai, 200001 China; 2 Department of Orthopaedic Surgery, Ninth People’s Hospital, Shanghai Jiaotong University School of Medicine, Shanghai, China

**Keywords:** Total knee arthroplasty, Morphometric parameter, Femoral trochlear groove, Morphological analysis, Computed tomography

## Abstract

**Background:**

Our study aimed to investigate whether geometrical features (size, shape, or alignment parameters) of the femoral condyle affect the morphology of the trochlear groove.

**Methods:**

Computed tomography models of 195 femurs (97 and 98 knees from male and female subjects, respectively) were reconstructed into three-dimensional models and categorised into four types of trochlear groove morphology based on the position of the turning point in relation to the mechanical axis (types 45°, 60°, 75°, and 90°). Only subjects with healthy knees were included, whereas individuals with previous knee trauma or knee pain, soft tissue injury, osteoarthritis, or other chronic diseases of the musculoskeletal system were excluded. The size parameters were: radius of the best-fit cylinder, anteroposterior dimension of the lateral condyles (AP), and distal mediolateral dimension (ML). The shape parameters were: aspect ratio (AP/ML), arc angle, and proximal- and distal- end angles. The alignment parameters were: knee valgus physiologic angle (KVPA), mechanical medial distal femoral angle (mMDFA), and hip-knee-ankle angle (HKA). All variables were measured in the femoral condyle models, and the means for each groove type were compared using one-way analysis of variance.

**Results:**

No significant difference among groove types was observed regarding size parameters. There were significant differences when comparing type 45° with types 60°, 75°, and 90° regarding aspect ratio and distal-end angle (*p <* 0.05), but not regarding proximal-end angle. There were significant differences when comparing type 90° with types 45°, 60°, and 75° regarding KVPA, mMDFA, and HKA (*p <* 0.05).

**Conclusion:**

Among size, shape, and alignment parameters, the latter two exhibited partial influence on the morphology of the trochlear groove. Shape parameters affected the trochlear groove for trochlear type 45°, for which the femoral condyle was relatively flat, whereas alignment parameters affected the trochlear groove for trochlear type 90°, showing that knees in type 90° tend to be valgus. The morphometric analysis based on trochlear groove classification may be helpful for the future design of individualized prostheses.

## Background

**Fig. 1:**
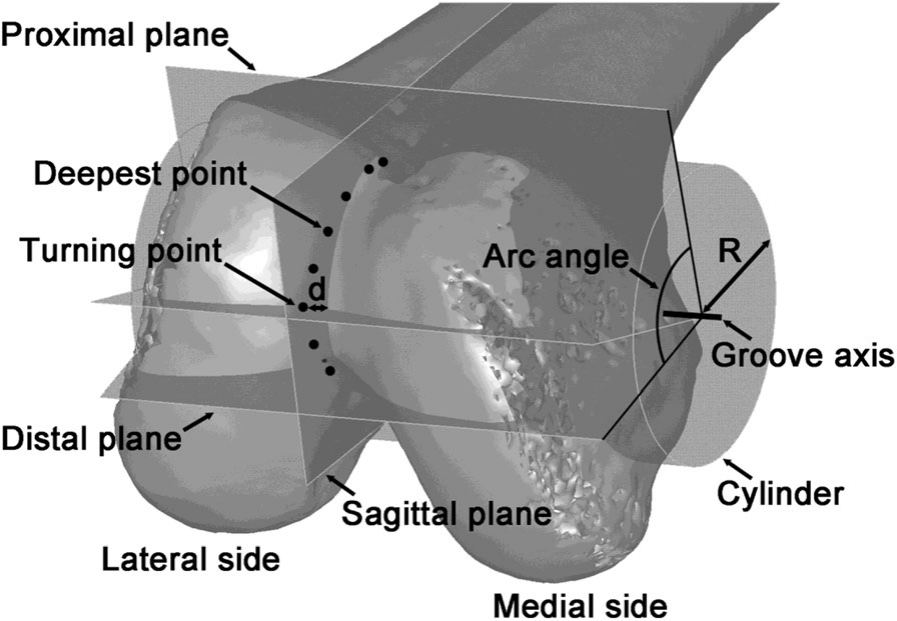
Methodology of classifying the trochlear groove tracking in terms of morphology. Three planes were established in three-dimensional models of the knee, constructed based on computed tomography data. A cylinder with its axis parallel to both the coronal and transverse planes was defined, and its radius was adjusted to allow the cylindrical surface to closely fit the trochlear groove. Coaxial cutting planes rotating in 15° increments (from 45° to 90°) about the trochlear groove axis (from the proximal to distal point) were defined. Subsequently, the deepest point of the trochlear groove was marked in each cross section. The distance from the turning point of the trochlear groove to the mechanical axis was measured (represented by d)

**Fig. 2:**
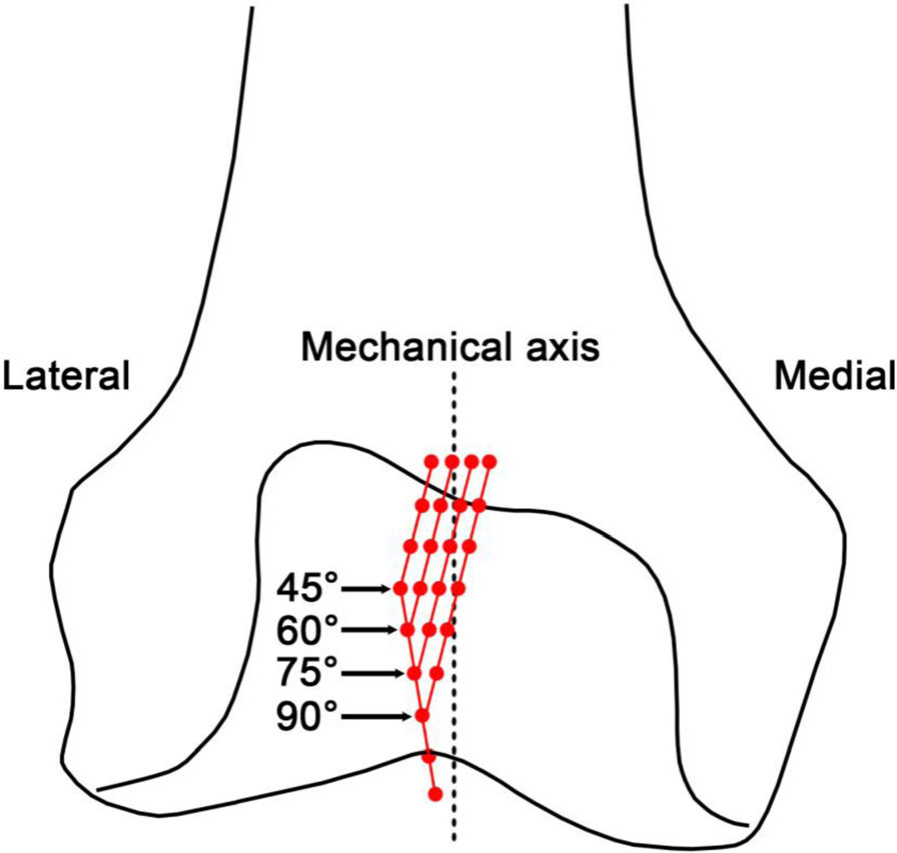
Description of the main components of knee morphology used in the present study. The trochlear groove was located laterally relative to the mechanical axis, and consisted of the laterally oriented proximal part and the medially oriented distal part. Based on the location of the turning points in different cross sections, the trochlear groove morphology was classified into four types (45°, 60°, 75°, and 90°). Adapted from ‘Morphological classification of the femoral trochlear groove based on a quantitative measurement of computed tomographic models’, Knee Surg Sports Traumatol Arthrosc, DOI: 10.1007/s00167-016-4236-5

Evaluating the morphology of the trochlear groove represents the theoretical foundation underlying the investigation of the differences between natural and prosthetic knees, or between natural and pathological knees. In a previous analysis of computed tomography (CT) femur models of 100 healthy Chinese subjects (200 knee models), the trochlear groove morphology was defined based on the position of the groove turning point relative to the femoral mechanical axis. Specifically, coaxial cutting planes were defined, rotated about the trochlear groove axis from the proximal to the distal end in 15° increments, and the deepest point of the trochlear groove was marked in each cross section (Figs. 1 and 2). Based on the location of the turning point in different cross sections, the trochlear groove was classified into four main types (45°, 60°, 75°, and 90°), which provided the theoretical fundamentals underlying anatomical category definitions in groove tracking [1]. This approach enables the development of better designed trochlear prosthetic components, optimally suited to diverse patient populations, and further provides the reference values to be used in the clinical setting for diagnosing patellofemoral diseases such as patellar maltracking and trochlear dysplasia.

However, it remains unclear whether there are certain geometrical features of the trochlear groove that lead to a specific type of trochlea groove morphology. This is a complex problem as there are many factors that may affect trochlear groove morphology. In a study by Lonner et al. [2], anthropomorphic differences regarding the size and shape of the distal femur were found between sexes, as the mean aspect ratio was larger for women than for men. In a study by Bellemans et al. [3], the shape of the knee was found to be influenced by both morphotype and sex, with sex accounting for 48% of the variability in distal femoral geometry, and morphotype accounting for 17%. Irrespective of sex, endomorph subjects had wider knees, whereas ectomorph subjects had narrower knees. Meanwhile, racial differences between Chinese and white populations in terms of knee size were reported in an anthropometric study by Yue et al. [4], with knees being generally smaller in Chinese than in white subjects. We wondered whether there are certain anatomical factors besides sex, morphotype, and racial factors that influence the shape of the distal femora, especially with respect to the trochlear groove. This is a crucial matter because morphological parameters of the condyle may help visualize the anatomical categories of the groove tracking and provide important information to be applied in individualized prosthesis design. Nonetheless, no study has been conducted to investigate the anatomical factors influencing the shape of the trochlear groove tracking in the context of the morphological classification based on the position of the groove turning point relative to the femoral axis.

Therefore, we hypothesized that, irrespective of sex and morphotype, certain morphological parameters in the femoral condyle itself could affect the shape of the trochlear groove tracking in Chinese individuals. The objective of our study was to investigate whether geometrical features (size, shape, or alignment parameters) of the femoral condyle affect the morphology of the trochlear groove.

## Methods

Our study enrolled the same sample population as that involved in the previous study described above (200 knee models from 100 Chinese subjects) [1]. Five cases were excluded because the knee models showed turning points located in the 30° and 105° cross sections. Finally, 195 knee models (97 knees from male subjects and 98 knees from female subjects) were included in the present analysis. Patients with healthy knees were included. The exclusion criteria were: (1) previous knee trauma or knee pain; (2) soft tissue injury; (3) osteoarthritis; (4) and other chronic diseases of the musculoskeletal system. The baseline characteristics of the included subjects, such as sex, age, height, weight, and body mass index (BMI), are listed in Table 1. There were no statistically significant differences regarding sex, age, height, weight, or BMI among the groups of patients classified according the four types of trochlear groove morphology.

**Table 1:** Baseline characteristics of the subjects whose knees were imaged and modelled for determining the geometrical features of the femoral condyle potentially relevant for the morphology of the trochlear groove

	Type 45°	Type 60°	Type 75°	Type 90°
Male, n	14	21	52	10
Female, n	6	26	51	15
Age, years	47.9 (30–57)	46.5 (33–57)	46.3 (31–60)	43.9 (30–60)
Height, cm	168.0 (156–182)	164.8 (150–179)	165.6 (150–181)	166.7 (153–190)
Weight, kg	66.1 (43–80)	65.2 (45–80)	65.5 (45–89)	68.2 (53–90)
BMI, kg/m^2^	23.3 (17.7–28.7)	24.0 (16.5–28.9)	23.9 (16.5–29.6)	24.5 (19.7–28.7

Parameter values are reported as count (n) or mean (range). BMI, body mass index

### Three-dimensional knee models

The CT examinations were not performed as part of our study. Instead, our study used the CT images employed in the previous study involving the same cohort [1]. The method of data collection applied in the previous study was as follows: CT scanning (Light Speed 16; GE Medical System, General Electric Company, Milwaukee, WI, USA) of both lower extremities was performed. To reduce radiographic exposure, the device was set to acquire CT slices at intervals of 0.625 mm for the knee joint, and at intervals of 2 mm for the hip and ankle joints (resolution 512 × 512 pixels). For each patient, the average effective radiation dose per scan was eight millisieverts, and iterative reconstruction was not utilized to reduce patient dose. Scanning data were then loaded into the Geomagic Studio 10.0 software (Geomagic Inc., Research Triangle Park, NC, USA) for three-dimensional reconstruction of the skeletal knee models.

### Establishment of three dimensional planes in the models

Before measurement, the three dimensional planes were established as follows: the mechanical axis was defined as the line connecting the centre of the femoral head to the apex of the intercondylar notch. The coronal plane was parallel to the mechanical axis and was externally rotated at 3° in relation to the line connecting the most posterior points of both femoral condyles. The sagittal plane was perpendicular to the coronal plane and passed through the mechanical axis at the midpoint of the two condyles. The transverse plane was perpendicular to both the coronal and sagittal planes.

### Classification of trochlear groove based on the turning point location in the cutting plane

A cylinder was established with its axis parallel to both the coronal and transverse planes, and its radius was adjusted to allow the cylindrical surface to closely fit the trochlear groove; its axis represented the trochlear groove axis. Coaxial cutting planes rotating about the trochlear groove axis from the proximal to distal end were created. A 0° cutting plane was represented by the plane parallel to the transversal plane. With 15° increments towards the distal end of the trochlear groove, eight cutting planes were created. The deepest points of the trochlear groove were marked at each cutting plane (Figs. 1 and 2). The trochlear groove tracking consisted of the laterally orientated proximal part and of the medially orientated distal part marked by the turning point. Based on the turning point location in the cutting plane, the trochlear groove was classified into type 45°, 60°, 75°, or 90° [1] (Figs. 1 and 2).

### Definitions of the morphometric parameters

**Fig. 3:**
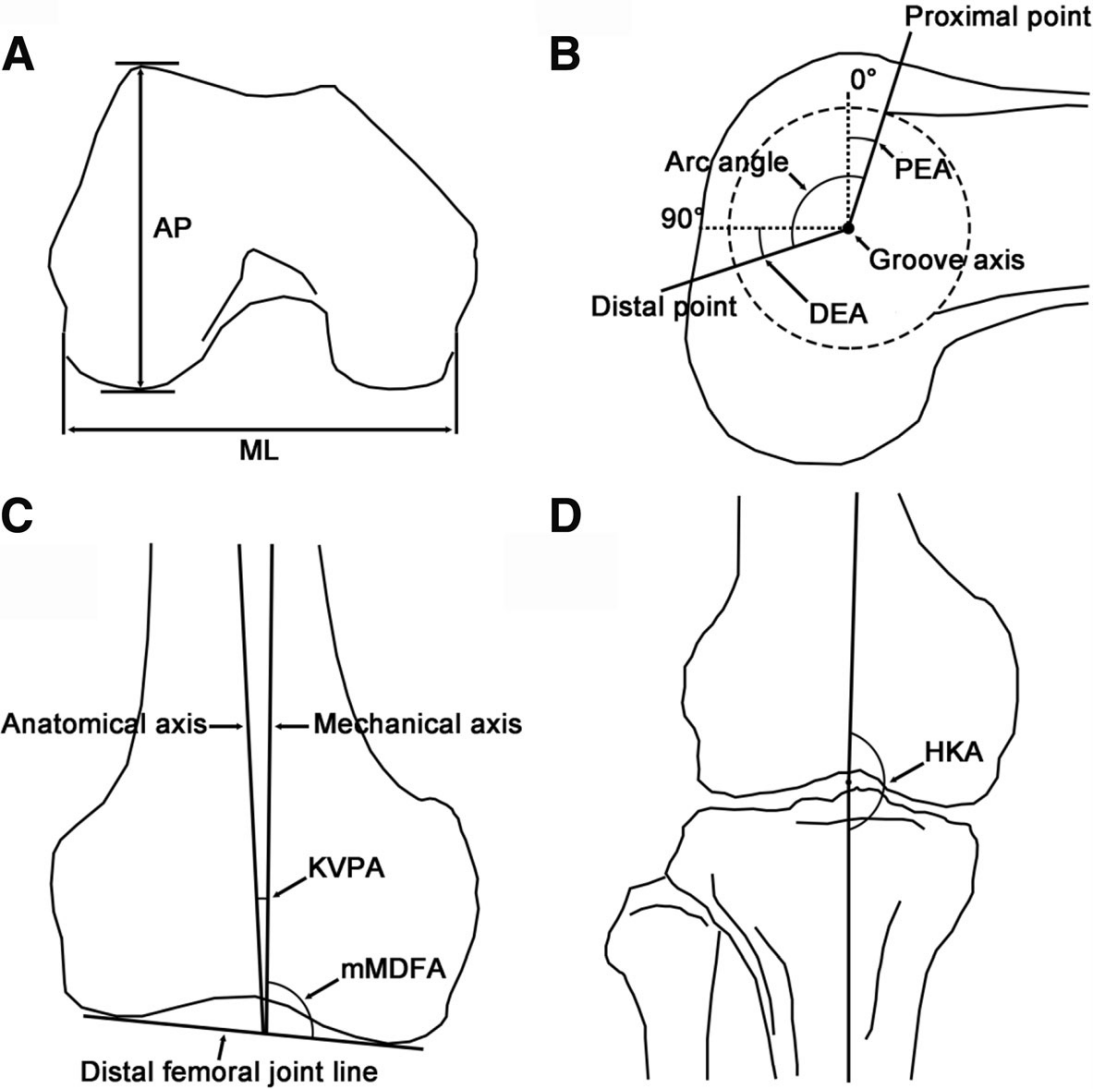
Definitions of the size-, shape-, and alignment-related parameters used in the present study. **a** AP, the anteroposterior dimension of the lateral condyles; ML, the femoral mediolateral dimension. **b** PEA, proximal-end angle; DEA, distal-end angle. **c** KVPA, the knee valgus physiological angle; mMDFA, mechanical medial distal femoral angle. **d** HKA, the hip-knee-ankle angle

The size parameters consisted of the radius of the best-fit cylinder (R), the anteroposterior dimension of the lateral condyles (AP), and the femoral mediolateral dimension (ML) (Figs. 1, 3a, and 3b) [5, 6]. The shape parameters consisted of the ratio AP/ML (aspect ratio), the angulation between the most proximal cutting plane and the most distal cutting plane (arc angle), the angulation between the most proximal cutting plane and the 0° cutting plane (proximal-end angle), and the angulation between the most distal cutting plane and the 90° cutting plane (distal-end angle) (Figs. 3a and 3b) [2, 5]. A negative angulation was defined for the cases in which the most proximal cutting plane was distal to the 0° cutting plane. The alignment parameters consisted of: the angle between the femoral mechanical axis and the femoral anatomical axis, defined as the knee valgus physiologic angle (KVPA); the tangent line to the distal femoral condyles (distal femoral joint line); the medial angulation between the femoral mechanical axis and the distal femoral joint line, defined as the mechanical medial distal femoral angle (mMDFA); and the medial angle between the femoral mechanical axis and the tibia mechanical axis, defined as the hip-knee-ankle angle (HKA). (Figs. 3c and 3d) [7–11].

A test-retest analysis was performed to determine intra-observer reliability, by measuring all parameters three times for 20 randomly selected femurs. The standard deviation of these three measurements was used to represent the accuracy of the measurement for each parameter.

### Statistical analysis

One-way analysis of variance was performed to determine if the anatomical parameters were significantly different among the groups classified by type of trochlear groove, as well as by age, height, weight, and BMI. The difference in sex ratio between each group pair was analysed using the Chi-square test. A *p*-value of less than 0.05 was considered statistically significant. This sample size was mainly chosen from the data in the pre-experiment of 42 knees and was calculated using a power analysis. The sample size for a power of ~ 0.9 (α = 0.05) indicated a minimum sample size of 48 knees.

## Results

The intra-observer reliability analysis indicated the following measurement accuracy for each of the evaluated parameters: 0.1 mm for R, AP, and ML; 0.1° for arc angle, as well as for the proximal- and distal-end angles; and 0.2° for KVPA, mMDFA, and HKA. The average values of the measurements are shown in Table 2. No significant difference was observed regarding R, AP, or ML. The average distal-end angle and aspect ratio for trochlear type 45° were 16.0° and 0.9, respectively. There were significant differences when comparing type 45° with types 60°, 75°, and 90° in terms of aspect ratio and distal-end angle (*p <* 0.05) (Table 3). No significant difference was found regarding the proximal-end angle. The mean arc angles were 104.6°, 107.9°, 107.8°, and 111.9° for types 45°, 60°, 75°, and 90°, respectively (Figs. 1 and 3), with significant difference being observed when comparing type 45° with type 90° or type 75° with type 90° (*p* < 0.05). The average values of KVPA, mMDFA, and HKA in type 90° were 4.4, 95.0°, and 181.3°, respectively, with significant difference being observed when comparing type 90° with types 45°, 60°, or 90° (*p* < 0.05). KVPA exhibited a decreasing trend, whereas mMDFA exhibited an increasing trend with the increase in the classified angle, from type 45° to 90°, sequentially (Table 4).

**Table 2:** Comparison of variables among different trochlear groove types

	R, mm	AP, mm	ML, mm
Type 45°	20.2 (1.7)	62.4 (4.0)	67.4 (5.9)
Type 60°	20.0 (1.5)	61.7 (3.7)	64.7 (4.8)
Type 75°	20.0 (1.7)	62.8 (4.0)	65.2 (5.3)
Type 90°	19.9 (1.8)	63.0 (4.5)	66.0 (5.5)
*p-*value	0.920	0.405	0.240

All parameter values are reported as mean (standard deviation)

*R* radius of the best-fit cylinder, *AP* anteroposterior dimension of the lateral condyles, *ML* femoral mediolateral dimension

**Table 3:** Comparison of shape parameters among different trochlear types

	Type 45°	Type 60°	Type 75°	Type 90°	*p*-value
Aspect ratio	0.9 (0.0)	1.0 (0.0)	1.0 (0.1)	1.0 (0.0)	p1* *=* 0.034
					p2* *=* 0.001
					p3* *=* 0.032
Arc angle,°	104.6 (6.3)	107.9 (8.2)	107.8 (8.2)	111.9 (8.4)	*p7* ^†^ *=* 0.003
					*p8* ^†^ *=* 0.030
Proximal-end angle,°	1.4 (5.7)	0.7 (5.7)	0.7 (4.7)	−1.9 (6.6)	0.126
Distal-end angle,°	16.0 (3.5)	18.6 (5.1)	18.5 (6.1)	20.0 (6.5)	p4* *=* 0.043
					p5* *=* 0.016
					p6* *=* 0.018

All parameter values are reported as mean (standard deviation)

Aspect ratio, the ratio between the anteroposterior dimension of the lateral condyles and the femoral mediolateral dimension; Arc angle, the angulation between the most proximal cutting plane and the most distal cutting plane; Proximal-end angle, the angulation between the most proximal cutting plane and the 0° cutting plane; Distal-end angle, the angulation between the most distal cutting plane and the 90° cutting plane

*There were significant differences when comparing type 45° with types 60° (*p1, p4*), 75° (*p2, p5*), and 90° (*p3, p6*) in terms of aspect ratio and distal-end angle, respectively

^†^There were significant differences when comparing type 45° with type 90° (*p7*), and type 75° with type 90° (*p8*) in terms of arc angle

**Table 4:** Comparison of alignment parameters among different trochlear types

	Type 45°	Type 60°	Type 75°	Type 90°	*p-*value
KVPA,°	5.1 (0.7)	5.1 (0.9)	5.1 (0.9)	4.4 (1.0)	p1* *=* 0.017
					p2* *=* 0.004
					p3* *=* 0.001
mMDFA,°	94.0 (1.2)	93.3 (2.1)	94.3 (2.0)	95.0 (1.6)	p4* *=* 0.031
					p5* *=* 0.008
					p6* *=* 0.001
HKA,°	179.8 (2.3)	179.3 (2.1)	179.6 (2.5)	181.3 (2.5)	p7* *=* 0.049
					p8* *=* 0.001
					p9* *=* 0.004

All parameter values are reported as mean (standard deviation)

*KVPA* knee valgus physiologic angle, *mMDFA* mechanical medial distal femoral angle, *HKA* hip-knee-ankle angle

*There were significant differences when comparing type 90° with types 45° (*p1, p4, p7*), 60° *(p2, p5, p8*), and 75° (*p3, p6, p9*) in terms of KVPA, mMDFA, and HKA, respectively

## Discussion

The most important finding in our study is that morphological parameters related to shape and alignment influence the morphology of the trochlear groove of type 45° and type 90°. These anatomical indicators may help in characterizing different types of trochlear groove tracking and provide useful information to be used in designing prosthetic components optimally suited for types 45° and 90°.

We performed a systematic assessment of the morphological parameters (size, shape, and alignment) of the femoral condyle in the context of each type of trochlear groove tracking. In a previous study [1], the distance between the turning point and the mechanical axis in the coronal plane was found to be 3.1, 2.8, 2.5, and 1.6 mm for types 45°, 60°, 75°, and 90°, respectively. However, no difference was found between the types of trochlear groove morphology in terms of ML, indicating that ML did not influence the shape of the trochlear groove. A similar conclusion was reached for AP and R in the sagittal plane. In our present study, we also found that the size-related parameters did not represent factors influencing the trochlear groove morphology, implying that there are four types of groove tracking in knee of various sizes. In terms of prosthesis design, four types of trochlear groove morphology should be taken into consideration when designing and sizing each femoral component.

On the other hand, the aspect ratio (AP/ML) and average distal-end angle were smaller in type 45° than in the other types (*p* < 0.05); moreover, the average value of the arc angle (Figs. 1 and 3) increased with the rotation of the coaxial cutting planes relative to the trochlear groove axis (104.6°, 107.9°, 107.8°, and 111.9° for types 45°, 60°, 75°, and 90°, respectively), and the value corresponding to type 45° was relatively small. These results suggest that the geometrical shape of the condyles represents a factor that influences the groove tracking morphology, and that the appearance of the trochlear condyle is relatively flat in type 45° compared to that in other types of groove morphology. Moreover, the rotation of the coaxial cutting planes relative to the trochlear groove axis was defined from the proximal to distal end (45° to 90°, at 15° intervals; Fig. 2), with the distance between the turning point and mechanical axis decreasing as the relative rotation increased [1]. Thus, the groove tracking in type 45° deviated from the mechanical axis to the highest extent. It has been reported that, as the measuring level moves distally along the femur, the sex-related deviation of the coronal width in the trochlear region increases progressively and peaks at the distal condyles [12]. Thus, it was speculated that the groove morphology of type 45° may be associated with relatively wider condyles, which is consistent with our present findings that the condyle is relatively flat, and the distal-end angle is smaller (*p* < 0.05) for type 45°. Furthermore, sexual dimorphism was previously reported in the morphology and shape of the distal femora [2]. Several studies have suggested that, for a given AP, ML tends to be wider in men than in women [3, 12–16]. In a study by Wang, et al. [5], the average arc angle in men was found to be 123.2°, which was smaller than that found in women (*p* < 0.05). As more than two thirds of the subjects with type 45° groove morphology were men, this may account for the decreased value of the aspect ratio (AP/ML) and arc angle we noted for type 45°.

We found that KVPA was significantly lower in type 90°, while mMDFA was significantly higher when comparing type 45° with type 90° or type 60° with 90° (*p* < 0.05). Furthermore, KVPA decreased, whereas mMDFA increased with the rotation of the coaxial cutting planes relative to the trochlear groove axis. It is possible that, as the turning points lies closer to the sagittal plane, the anatomical axis rotates towards the mechanical axis, and the distal femoral line rotates towards a more pronounced valgus position, away from the mechanical axis, which results in decreased KVPA and increased mMDFA. The HKA was significantly higher in type 90° (*p* < 0.05). In a study by Wang et al. [7], the HKA was shown to exhibit a significant, inverse correlation with the KVPA. Furthermore, an increased HKA angle (more pronounced valgus knee) was correlated with an increased mMDFA, as reported by Zeng, et al. [8], indicating that the knee with type 90° groove morphology tends to be in valgus. Moreover, a slightly more valgus alignment of the knee was found in women [8]. In our study, 60% of subjects with type 90° groove morphology were women, which may account for the more pronounced tendency for valgus knee.

Importantly, the shape- and alignment-related parameters were two main factors affecting the morphology of the trochlear groove. The trochlear groove was classified into types 45° (10.0%), 60° (23.5%), 75° (51.5%), and 90° (12.5%) in the previous study [1]. The groove tracking with morphology of types 45° and 90° (22.5%) was affected by such parameters, while the remaining groove morphology types (75%) were hardly affected. Therefore, a wider design for prosthetic components should be considered for knees with type 45° groove morphology (10%), while prostheses with type 90° morphology (12.5%) may be more suitable in patients with valgus knee.

The main limitation of our study is that using data obtained via CT measurements, which lies at the core of our approach, implies ignoring the cartilage layer that covers the trochlear surface of the femur. While the geometry of the cartilage surface is known to differ from that of trochlear bone, the difference is relatively small [1, 17, 18], and thus we do not expect this aspect to affect our conclusions. The other limitation of our study is that our findings reflect the relationships between condyle parameters and trochlear groove morphology only in healthy adults. Therefore, further study is warranted to clarify this relationship in arthritic knees, which represent a common target for prosthetic implants.

## Conclusions

Our study involving Chinese individuals with healthy knees revealed that, among the size-, shape-, and alignment-related parameters, the latter two showed a partial influence on the morphology of the trochlear groove. Shape-related parameters affected the morphology of trochlear groove type 45°, where the femoral condyle is relatively flat. Alignment-related parameters affected the morphology of trochlear groove type 90°, where the knee tends to be in valgus. Such observations from the morphometric analysis based on the trochlear groove classification may provide useful information for individualized prosthesis design in Chinese populations.

## Abbreviations

AP: Anteroposterior dimension of the lateral condyles
AP/ML: Aspect ratio
BMI: Body mass index
CT: Computed tomography
HKA: Hip-knee-ankle angle
KVPA: Knee valgus physiologic angle
ML: Distal mediolateral dimension
mMDFA: Mechanical medial distal femoral angle
